# Does the Association Between Healthy Lifestyle and Cardiometabolic Variables in Adolescents Depend on Obesity and Its Distribution?

**DOI:** 10.3390/healthcare14030328

**Published:** 2026-01-28

**Authors:** Tiago Rodrigues de Lima, Mateus Augusto Bim, Andreia Pelegrini, Diego Augusto Santos Silva

**Affiliations:** 1 Health and Sports Sciences Center, Department of Physical Education, State University of Santa Catarina, Florianópolis 88040-900, Brazil; mateus.gepecin@gmail.com (M.A.B.); andreia.pelegrini@udesc.br (A.P.); 2 Research Center in Kinanthropometry and Human Performance, Department of Physical Education, Sports Center, Federal University of Santa Catarina, Florianopolis 88035-972, Brazil; diegoaugustoss@yahoo.com.br

**Keywords:** alcohol drinking, behavior, cardiovascular diseases, diet, exercise, smoking

## Abstract

**Background/Objectives**: The present study aimed to examine how obesity and its distribution influence the relationship between healthy lifestyle habits and cardiometabolic health indicators in adolescents. **Methods**: This cross-sectional study included 340 adolescents (54.8% female; mean age, 16.6 ± 1.0 years) from Brazil. The cardiometabolic variables included systolic (SBP) and diastolic blood pressure (DBP), high-sensitivity C-reactive protein (CRP), and markers of lipid and glucose metabolism. Information on regular physical activity, healthy diet, reduced alcohol consumption, and non-smoking was collected via a self-reported questionnaire. Body mass index, waist circumference, and skinfold measurements were assessed to determine general obesity, abdominal obesity, and excess body fat, respectively. Multiple linear regression, adjusted for confounding factors, was employed for the analysis. **Results**: The adoption of ≥3 healthy lifestyle habits was directly associated with high-density lipoprotein cholesterol (up to 1.2 mg/dL) and inversely associated with triglycerides (up to −0.11 p.p.). Engaging in multiple healthy lifestyle habits was inversely associated with SBP among adolescents with general (*p* = 0.018) and central obesity (*p* = 0.004). Furthermore, the adoption of multiple healthy lifestyle habits was inversely associated with CRP in adolescents with central obesity (*p* = 0.037). **Conclusions**: Even in adolescents with obesity, it is speculated that the adoption of healthy habits may contribute to a reduction in cardiometabolic risk, given the inverse association with SBP in those with general and central obesity and the inverse association with CRP in adolescents with central obesity.

## 1. Introduction

A healthy lifestyle can be defined as a multidimensional pattern of behaviors, including regular physical activity, balanced nutrition, and the avoidance of harmful substances like tobacco and alcohol, which collectively promote physical and mental well-being and reduce the risk of non-communicable diseases. In adolescence, the consolidation of these behaviors is critical, as they tend to persist into adulthood. Lifestyle plays a fundamental role in the development and exacerbation of cardiometabolic risk [1]. Regular physical activity is directly associated with a reduction in inflammatory states and improvements in hemodynamic health in children and adolescents with obesity [2]. An appropriate diet involves reducing the intake of foods linked with increased cardiometabolic risk. This includes limiting ultra-processed foods high in sodium and saturated and trans fats while simultaneously increasing the consumption of fruits and vegetables. These foods, rich in antioxidants, help prevent lipid deposition in the arteries [3]. While the cardioprotective effects of alcohol are controversial in youth, evidence suggests that even low consumption in adolescence can alter peripheral vascular resistance and contribute to early arterial remodeling [4]. Furthermore, smoking cessation is a key recommendation for reducing cardiometabolic risk [3].

Childhood obesity remains a global health crisis, with over 390 million children and adolescents aged 5 to 19 classified as overweight in 2022, including 160 million who were affected by obesity [5]. Obesity plays a determining role in cardiometabolic health. Adipocytokines secreted by adipose tissue are directly associated with damage to the vascular system, glycemic metabolism, and low-grade inflammation, which predisposes individuals to atherosclerosis [6]. However, given its heterogeneous nature [7], it is hypothesized that the adverse effects of obesity on cardiometabolic health may be more closely related to individual variations in regional fat distribution. Specifically, it is expected that the presence of high-grade inflammation and increased oxidative stress characteristic of adolescents with obesity may act as a biological modifier. This means that the systemic benefits typically derived from healthy lifestyle habits, such as improved endothelial function or lipid clearance, might be partially blunted in the presence of excess adiposity. Conversely, these habits may be more critically necessary to overcome the elevated baseline cardiometabolic risk [8,9].

For epidemiological purposes and routine clinical practice, simple anthropometric measurements are commonly used as screening tools for obesity. The body mass index (BMI) serves as an indirect measure of body fat in children and adolescents, with its classification based on growth curves adjusted for sex and age [10]. Waist circumference is employed to assess abdominal or central obesity, which is directly linked to increased cardiometabolic risk in children and adolescents [11]. These risk factors increase the likelihood of developing diabetes or experiencing future vascular events. Additionally, body fat percentage (%BF), estimated through skinfold measurements, is another metric used to assess general adiposity and has a strong association with cardiometabolic variables [7].

Although adopting multiple healthy lifestyle habits is a critical factor in reducing cardiometabolic disease risk in adolescents [1,11], there is limited understanding of whether improvements in cardiometabolic indicators (e.g., blood pressure, lipids, biomarkers of glucose metabolism, and low-grade inflammation) occur when adolescents with obesity adopt different healthy lifestyle habits [1]. Young individuals with overweight or obesity are approximately five times more likely to experience excessive adiposity in adulthood. This transition is typically accompanied by a heightened risk of obesity-related comorbidities [1]. These comorbidities, such as cardiovascular diseases (e.g., heart attack, stroke), type 2 diabetes, insulin resistance, and non-alcoholic fatty liver disease, represent the substantial burden of the obesity epidemic on public health [11].

Investigating the role of obesity in different regions of the body concerning healthy lifestyle habits and cardiometabolic indicators enables us to assess whether the anticipated health benefits resulting from adopting a healthy lifestyle are consistent across adolescents or influenced by specific patterns of obesity. The findings of this study are crucial for identifying whether adolescents with localized obesity require tailored interventions to optimize the adoption of healthy behaviors, particularly when aiming to mitigate cardiometabolic risks. This line of inquiry holds particular significance for low- and middle-income countries, where limited resources for health investments [12] make it essential to develop cost-effective and evidence-based strategies. By providing a deeper understanding of the interplay between lifestyle habits and cardiometabolic health in adolescents, especially those with obesity, this research can inform the design of practical interventions at various levels, such as primary health care, a cornerstone for advancing towards universal health coverage.

Therefore, the objective of the present study was to investigate whether obesity moderates the relationship between healthy lifestyle habits and cardiometabolic health indicators in adolescents. We hypothesize that the association between healthy lifestyle habits and cardiometabolic health indicators will be moderated by obesity and its distribution.

## 2. Materials and Methods

### 2.1. Study Design

This cross-sectional study uses data from the “*Guia Brasileiro de Avaliação da Aptidão Física Relacionada à Saúde e Hábitos de Vida—Etapa II*” project, a population-based investigation designed to examine the relationship between health-related physical fitness indicators (e.g., muscular strength, body composition) and clinical, blood, and lifestyle variables among school-aged adolescents. Conducted in the second semester of 2019, the project included a representative sample of adolescents aged 14 to 19 years, enrolled in public high schools in São José, Southern Brazil.

The study received approval from the Ethics Committee on Human Research at the Federal University of Santa Catarina (protocol n° 3.523.470). All participating adolescents were required to provide a signed Assent Form, along with a Free and Informed Consent Form from their guardians (age < 18 years) or from themselves (age ≥ 18 years). Individuals with physical disabilities that limited their participation in physical assessments were excluded from the study.

### 2.2. Study Population and Sampling

**Figure 1:**
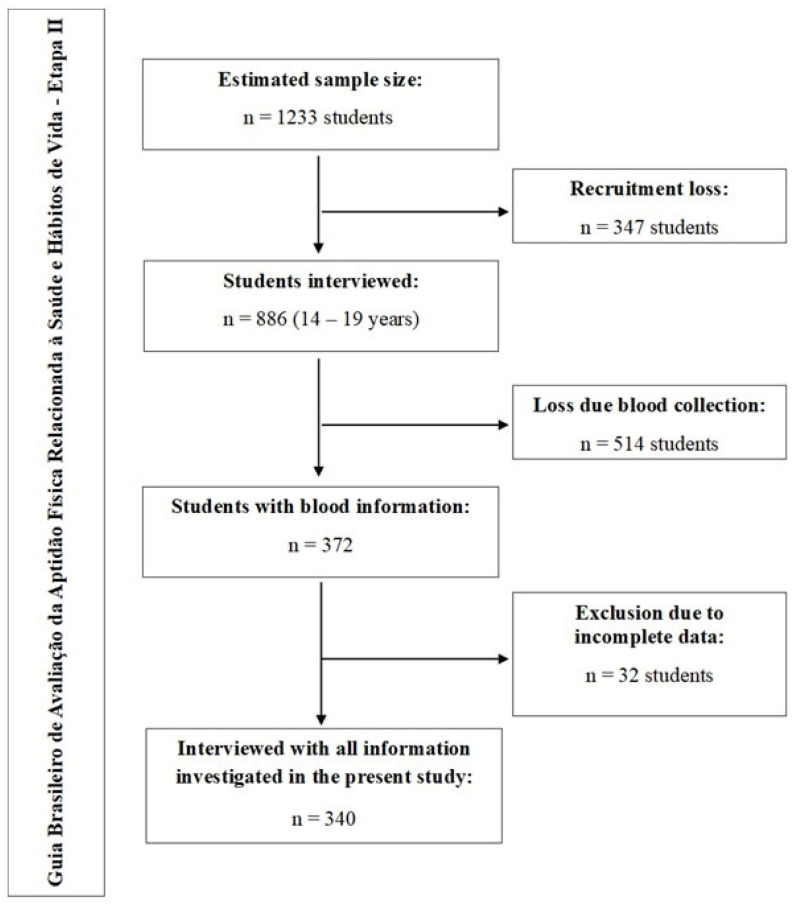
Flowchart of Guia Brasileiro de Avaliação da Aptidão Física Relacionada à Saúde—Etapa II—and the variables used in the study.

Sampling of the “*Guia Brasileiro de Avaliação da Aptidão Física Relacionada à Saúde e Hábitos de Vida—Etapa II*” project was performed in two stages: (1) stratified by public high schools (according to density—11 eligible public schools in São José) and (2) clustered by classes considering school shift and grade (186 high school classes—77.1% of students were on the morning shift). Given that 5411 students (14–19 years old) were enrolled for the 2019 school year, and using a confidence level of 1.96 (95% confidence interval), a tolerable error of five percentage points, a prevalence estimate of 50%, a design effect of 1.5, and an additional 20% to account for possible losses and refusals, as well as another 20% to control for potential confounders, the calculated required sample size was 1233 students. Following the recruitment phase, 886 adolescents were interviewed. Due to budgetary constraints and the timing of authorization returns, a sub-sample of 372 students underwent blood collection. After excluding 32 participants with missing data across the investigated variables, a final analytical sample of 340 students was achieved (Figure 1).

While this final analytical sample is smaller than the initial target for population representativeness, a sensitivity power analysis was conducted to ensure the internal validity of the moderation tests. The calculation of the available statistical power is important as it helps determine whether the study is worthwhile in terms of its conduct or whether the results of the analyses conducted provide accurate results or not [13]. For the determination of the available statistical power, the statistical modeling used (multiple linear regression), the number of predictor variables, and the adjustment variables (sociodemographic variables and sexual maturation) for these models were considered. In this context, the number of students necessary to reject the null hypothesis in the investigations varied from 126 to 179 individuals, based on a medium effect size (f^2^ = 0.15) and the desired power (1-β = 0.95). Thus, the sample of 340 provides robust statistical support for the interaction analyses, although we acknowledge the impact of the attrition rate on the generalizability of the findings.

### 2.3. Measurement Instruments

#### 2.3.1. Cardiometabolic Profile

Systolic blood pressure (SBP) and diastolic blood pressure (DBP), high-sensitivity C-reactive protein (CRP) levels, and markers of lipid and glucose metabolism were used in this study.

SBP and DBP were measured following established literature recommendations [14], using an oscillometric method with a calibrated Omron (Kyoto, Japan) electronic device, model HEM 742. Each adolescent underwent two measurements, with a rest period of at least 15 min before and between measurements. If the difference between the two readings exceeded 10 mmHg for either systolic or diastolic blood pressure, a third measurement was conducted, with the highest value being replaced. The mean of the two measurements was used for each variable.

Venous blood samples were collected by nurses in the school environment during the early morning, following a fasting period of at least 8 h. CRP levels (mg/L) were quantified using a turbidimetric method. The lipid profile (cholesterol—CHOL (mg/dL); triglycerides—TRGs (mg/dL); HDL-C (mg/dL); low-density lipoprotein cholesterol—LDL-C (mg/dL)), fasting glucose (FG, mg/dL), and fasting insulin (FI, mU/L) were determined using a colorimetric test. The homeostatic model assessment of insulin resistance (HOMA-IR) was calculated as described previously [15] using the following formula: HOMA-IR = (FG × 0.0555 × FI)/22.5. Due to their non-normal distribution, TRG, CRP, and HOMA-IR values were transformed to their natural logarithm (ln).

#### 2.3.2. Healthy Lifestyle

Data on general lifestyle habits (physical activity, alcohol consumption, smoking, and dietary habits) were collected. Physical activity and alcohol consumption were assessed using questions from the Brazilian version of the Youth Risk Behavior Surveillance System (YRBSS), used in the United States, translated and validated for Brazil, with a moderately high agreement rate, with an average of 68.3% and a median of 68.5% [16]. Smoking and diet habits were evaluated using the Fantastic Lifestyle questionnaire, developed by the Canadian Society for Exercise Physiology [17], translated and validated in Brazil, demonstrating construct validity with a classification capacity of 75.0% and 80.7%, Kappa indices of 0.58 and 0.70, and good reproducibility with a high correlation between instrument classes (R = 0.92; *p* = 0.2) [18]. Diet habits were assessed with the Fantastic Lifestyle questionnaire, which asks participants to consider their typical behaviors over the past 30 days. This instrument provides a global assessment of lifestyle patterns rather than precise caloric or nutrient quantification. The multidimensional assessment of lifestyle was achieved through a strategic combination of selected items from the YRBSS and the Fantastic Lifestyle questionnaire, both of which are validated for the Brazilian context [16,18]. These instruments were integrated into an ordinal composite scale (0 to 3+), a method grounded in the “cumulative health behavior” theory. This approach posits that the co-occurrence of healthy habits produces a synergistic physiological effect on the vascular and metabolic systems that exceeds the sum of individual behaviors [19]. By focusing on the cumulative impact rather than isolated practices, this composite scale captures the broader lifestyle profile and reduces the measurement noise inherent in individual self-reported questions.

Physical activity was assessed with the following question: during the past 7 days, on how many days were you physically active for at least 60 min a day (consider the time you spent in any kind of physical activity that increased your heart rate and made your breathing faster for some time)? [16]. Adolescents who reported engaging in physical activity for at least 60 min on all seven days a week were classified as meeting the physical activity recommendations [20]. For those aged 18 years and older, compliance with physical activity recommendations was defined as performing at least 150 min of moderate-intensity physical activity per week, at least 75 min of vigorous-intensity physical activity per week, or an equivalent combination of moderate- and vigorous-intensity activities [20]. In this context, questions regarding the weekly frequency (0, 1, 2, 3, 4, 5, 6, 7 days) and daily duration (0—does not practice; 1—less than 30 min per day; 2—30 to 59 min per day; 3—60 min or more per day) of each session of moderate- to vigorous-intensity physical activity in a typical week, as well as the number of times per week that the participant was moderately active OR vigorously active (0—less than once; 1—1 to 2 times; 2—3 times; 3—4 times; 4—5 or more times), were utilized.

The question regarding alcohol consumption was as follows [16]: “During the last 30 days, on how many days did you consume five or more alcoholic drinks in a single occasion? (A serving is defined as one can of beer, a glass of wine, a shot of whiskey, rum, vodka, etc.)” Participants who answered negatively to this question were considered to have adopted a healthy habit concerning alcohol consumption.

Dietary habits were assessed using the following questions related to a typical week [18]: “Do you follow a balanced diet?” A balanced diet was considered to include grains and cereals (5 to 12 servings per day); fruits and vegetables (5 to 10 servings per day); meats and similar products (2 to 3 servings per day); and milk and dairy products (3 to 4 servings until the age of 16 and 2 to 4 servings above the age of 16). Response options included the following: almost never, rarely, sometimes, with relative frequency, and often. Participants who answered “with relative frequency” or “often” were classified as exhibiting healthy dietary habits.

Smoking habits were assessed using the following question [17]: “Do you smoke cigarettes?” The response options for this question were as follows: (0) never smoked; (1) 1 to 10 cigarettes per day; (2) more than 10 cigarettes per day; (3) none in the last six months; and (4) none in the last year. Given the absence of a safe level for health regarding cigarette use [21], adolescents who responded that they had never used cigarettes were classified as having a healthy habit concerning cigarette use. Adolescents who answered positively to any of the other questions were classified as not exhibiting appropriate behavior regarding cigarette use.

The number of healthy lifestyle habits (physically active, balanced diet, low alcohol consumption, and non-smoking) was summed and converted into an ordinal scale ranging from 0 (no healthy lifestyle components) to 4 (all components). Due to the low prevalence of adolescents adopting all four healthy habits (0.88%; n = 3), the categories for individuals adopting 3 or 4 healthy habits were combined. Therefore, for analytical purposes, the scale was regrouped into 0 points, 1 point, 2 points, and 3+ points.

#### 2.3.3. Obesity

Anthropometric measurements were performed by a trained team of undergraduate and graduate students. Before data collection, all evaluators underwent standardization training based on the International Society for the Advancement of Kinanthropometry (ISAK) guidelines [22]. Quality control was assessed using the Technical Error of Measurement (TEM), comparing evaluators’ results with a gold-standard ISAK-certified anthropometrist. The maximum inter-evaluator TEM was 0.33%, and the intra-evaluator TEM was 0.23%, indicating an adequate level of reliability [23]. Body fat was estimated using the triceps and subscapular skinfolds according to the Slaughter et al. [24] equation, which is specifically validated for the pediatric population.

The anthropometric variables assessed included height, body mass, and waist circumference, with the mean of two measurements for each variable used in the analyses [22]. Height was measured using a Sanny^®^ stadiometer (Sao Paulo, Brazil), and body weight was recorded with a G-tech^®^ digital scale (Zhongshan, China). Waist circumference was measured at the narrowest part of the trunk with an anthropometric tape (Sanny^®^, Sao Paulo, Brazil). Waist circumference values were classified concerning abdominal obesity based on the cut-points proposed by Taylor et al. [10], defining excess abdominal fat as values with a z-score ≥ 1 [10]. These cut-points are approximately equivalent to the 85th percentile, which is used for classifying overweight using BMI [10]. The cut-points were age- and sex-specific, with sensitivity and specificity values of 84% and 94% for females and 87% and 92% for males, respectively [10]. The BMI was initially estimated as a continuous variable (kg/m^2^) and classified based on World Health Organization (WHO) cut-points, which define overweight as >1 standard deviation and obesity as >2 standard deviations from the mean [25]. Given the low prevalence of obesity (8.5%), participants with overweight and obesity were combined into a single “excess weight” category (BMI for age > +1 SD). This grouping was methodologically necessary to avoid the “sparse-cell” problem in interaction modeling, which would otherwise lead to unstable point estimates and reduced precision in the assessment of the moderating effect. This approach allows for robust comparison between adolescents facing an increased metabolic load from excess adiposity and their normal-weight counterparts.

The body fat percentage (%BF) was determined using measurements of triceps and subscapular skinfolds. These measurements were conducted following the guidelines set by ISAK [22]. The %BF was estimated using the Lohman predictive equation [26], as follows: %BF = 1.35 × (triceps skinfold + subscapular skinfold) − 0.012 × (triceps skinfold + subscapular skinfold)^2^ − Intercept. The intercept, also referred to as the constant, varies by sex and age. In this study, we used constants suggested by Pires-Neto and Petroski [27], who adapted Lohman’s equation specifically for Brazilian children and adolescents [26]. Consequently, the constants differed by sex, age, and self-reported ethnicity/race (white, brown, black, yellow, and indigenous), following the Brazilian Institute of Geography and Statistics (IBGE) standards [27,28]. This adaptation is essential to improve the accuracy of fat-free mass estimation (used in determining %BF), accounting for ethnic-related variations in body density, a critical factor for the internal validity of body composition assessment in highly admixed populations like the Brazilian one.

After calculating the %BF, adolescents were classified based on body fat distribution using cut-points suggested by FITNESSGRAM^®^ [29]. This classification is divided into sex- and age-specific categories: Healthy Fitness Zone (HFZ), Needs Improvement (NI), and Needs Improvement–Health Risk (NI-HR). The HFZ represents the ideal range for sex- and age-appropriate %BF values [29]. Adolescents classified in the NI and NI-HR zones did not meet the FITNESSGRAM^®^ health-related fitness standards [29] and were categorized as having excess body fat.

Sociodemographic variables and maturational status were included as control variables in the multivariate models. Sociodemographic variables included sex (male/female), age (in years), and socioeconomic status. Socioeconomic status was assessed according to the Economic Classification Criteria of Brazil, established by the Brazilian Association of Market Research Companies [30], using a questionnaire that evaluates the purchasing power of adolescent families. Questions regarding the presence and quantity of household appliances in general (bathroom, personal computer, DVD player, microwave, clothes dryer, dishwasher, refrigerator and freezer, washing machine, car, motorcycle) or monthly-paid workers in the household are used in this assessment. The total score after summing the points assigned to each response can vary from 0 to 100 points, distributed among the following classes: A, B1, B2, C1, C2, and D + E [30]. The higher the score, the higher the economic level.

Maturational status was assessed using Tanner’s criteria [31] through self-assessment figures depicting stages of sexual maturation, as adopted in a sample of Brazilian schoolchildren [32]. The assessment included breast development and pubic hair for females and genital development and pubic hair for males. For the present study, the lowest values for pubic hair in both males and females, as well as breast development (female) and genital development (male), were used in the analyses. Tanner’s stages are categorized as follows: Stage 1 represents the pre-pubertal stage; Stages 2, 3, and 4 represent puberty; and Stage 5 indicates post-pubertal status. The adolescents in the present study were classified in the pre-pubertal, pubertal, and post-pubertal categories [31,32].

### 2.4. Statistical Analysis

All analyses accounted for sampling weights and the survey design. Data analysis was conducted using Stata 16.0 (StataCorp LP, College Station, TX, USA). Symmetric continuous variables were summarized using the mean and standard deviation, while asymmetric variables were described using the median and interquartile range (25th–75th percentiles). Categorical variables were presented as percentages (%). Depending on the nature of the investigated variables, the χ^2^ (chi-square) test or *t* test for independent samples was used to identify possible differences according to sex.

Various multiple linear regression models were employed to examine the relationship between the number of healthy lifestyle habits—exposure variables (0, 1, 2, 3+ variables)—and each cardiometabolic indicator (SBP, DBP, CHOL, HDL-C, LDL-C, TRGs, FG, HOMA-IR, and CRP). For each of the models analyzed, the sex, age, socioeconomic status, and sexual maturation variables were included as covariates. The results are presented as regression coefficients (β) with their respective standard error (SE). For lnCRP, lnHOMA-IR, and lnTRG, the results were back-transformed to the exponential form (_EXP_β) and should be interpreted as a risk ratio relative to the mean value observed among those who did not adopt any of the healthy lifestyle habits.

The potential moderating role of obesity was assessed by including interaction terms (i.e., general overweight/obesity, abdominal obesity, or excess of body fat) in the regression models, analyzing the association between healthy lifestyle habits and cardiometabolic variables. A *p* value < 0.10 for the interaction term was considered indicative of the heterogeneity of the associations [33]. This threshold was adopted because statistical tests for heterogeneity/interaction are known to have limited power; therefore, as suggested in the literature, a higher *p* value than usual (i.e., *p* < 0.10) is a more appropriate and sensible cut-off for these analyses [33]. Partial eta-squared (η^2^) was used as a measure of the effect size of the interaction results. Small, medium, and large effects were reflected in values of η^2^ equal to 0.0099, 0.0588, and 0.1379, respectively [34]. Predicted adjusted means for the outcomes across different categories of the healthy lifestyle habits scale, stratified by the presence or absence of general overweight/obesity, abdominal obesity, or excess body fat, were estimated and are presented graphically. All models were adjusted for sex and age as covariates in addition to sociodemographic and maturational status variables. Preliminary analyses tested for sex and age interactions. However, since no significant moderating effects were found for these variables, they were maintained solely as adjustment factors to control for potential confounding, without further stratification.

## 3. Results

The present study investigated data from 340 schoolchildren aged 14 to 19 years in São José, Santa Catarina, Brazil. General information on the entire sample and according to sex is available in Table 1. In general, females presented higher mean values for waist circumference, body fat, and presence of overweight/obesity compared to males (*p* < 0.05). Conversely, males were older and presented a higher prevalence of adopting healthy dietary habits and physical activity when compared to females (*p* < 0.05) (Table 1). Regarding healthy lifestyle habits, when stratifying the results according to age groups (14–17 years/18–19 years), a higher prevalence of compliance with physical activity recommendations was identified in those evaluated aged 18–19 years when compared to those aged 14–17 years (*p* < 0.05). For other healthy lifestyle habits, no differences were identified between age groups (results not presented).

**Figure 2:**
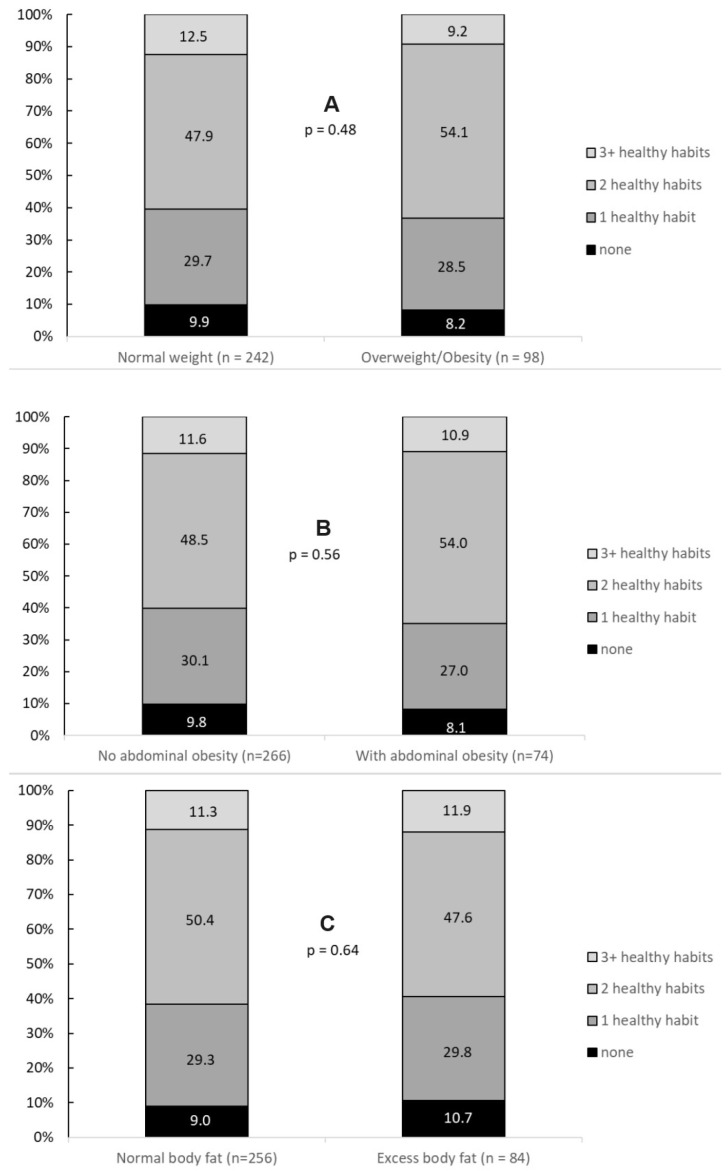
Prevalence of healthy lifestyle habits according to the presence of general obesity (**A**), abdominal obesity (**B**), and excess body fat (**C**).

Regardless of the prevalence of obesity, approximately one in ten adolescents adopted ≥ 3 lifestyle habits (Figure 2). Additionally, no significant differences were observed in the adoption of lifestyle habits based on the presence of general obesity (Figure 2A), abdominal obesity (Figure 2B), or excess body fat (Figure 2C).

The adoption of healthy lifestyle habits was positively associated with HDL-C and negatively associated with lnTRG (*p* < 0.05). Additionally, significant interactions (*p* < 0.10) were observed between healthy lifestyle habits and obesity indicators for SBP, CHOL, lnTRG, LDL-C, and lnCRP. Specifically, general obesity modified the association with SBP (η^2^ = 0.015) and LDL-C (η^2^ = 0.014). Abdominal obesity modified the associations with SBP (η^2^ = 0.019), CHOL (η^2^ = 0.022), and lnCRP (η^2^ = 0.015). Body fat interacted with healthy lifestyle habits for lnTRG levels (η^2^ = 0.005) (Table 2). According to the adopted criteria, these values reflect a small effect size for the moderating role of adiposity in the tested models. Graphical results for the identified interactions can be seen in Supplementary Figures S1–S6.

Detailed information regarding the adjusted results of the association between healthy lifestyle habits and cardiometabolic indicators, as well as the intercept values for the interactions between obesity and healthy habits when associated with cardiometabolic indicators—along with the respective 95% confidence intervals, *p* values, and effect size values for each of these outcomes—can be seen in Supplementary Tables S1–S3.

**Figure 3:**
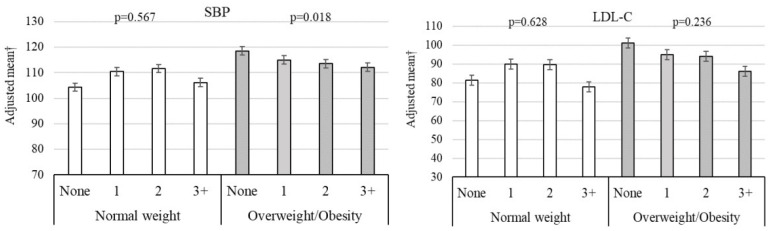
Adjusted association between cardiometabolic variables and presence of overweight/obesity, stratified by healthy lifestyle components. SBP, systolic blood pressure; LDL-C, low-density lipoprotein cholesterol.

**Figure 4:**
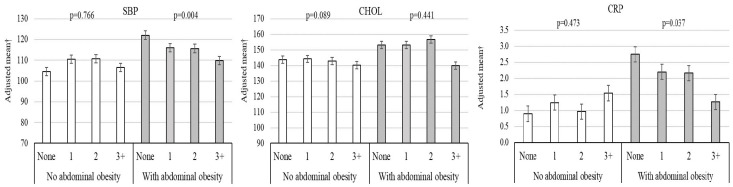
Adjusted association between cardiometabolic variables and presence of abdominal obesity, stratified by healthy lifestyle components. SBP, systolic blood pressure; CHOL, cholesterol; CRP, high-sensitivity C-reactive protein.

**Figure 5:**
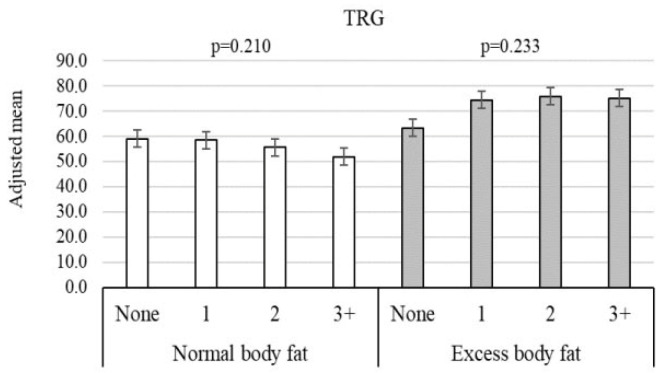
Adjusted association between cardiometabolic variables and presence of excess body fat, stratified by healthy lifestyle components. TRGs, triglycerides.

Heterogeneity results for the interaction between the outcomes analyzed and the number of lifestyle habits as well as the presence of general obesity, abdominal obesity, and excess body fat are presented in Figure 3 and Figure 4 and Figure 5, respectively. Adopting a greater number of healthy lifestyle habits showed a cumulative inverse association with SBP among individuals with overweight/obesity (Figure 3). An inverse association between lifestyle habits and both SBP and lnCRP was observed among individuals with abdominal obesity (Figure 4).

The potential moderating role of adiposity was further explored through predicted interaction models (Figure 3, Figure 4 and Figure 5). While point estimates in intermediate categories sometimes show overlapping 95% CIs, Supplementary Figures S1–S6 provide a detailed decomposition of these interactions. Specifically, Supplementary Figures S1, S2 and S5 illustrate a clear divergence in cardiometabolic trajectories, where the benefit of accumulating healthy habits (3+ habits vs. none) is significantly magnified in the presence of general or abdominal obesity. These visualizations support the findings in Table 2, showing that the most substantial risk reductions are concentrated in the highest-risk adiposity subgroups.

Although the heterogeneity results suggested a potential moderating effect of body fat on the relationship between healthy lifestyle and TRGs, no significant differences were found in TRG levels based on the number of lifestyle habits adopted (Figure 5).

## 4. Discussion

The present study aimed to explore the potential moderating effect of general obesity, central obesity, and excess body fat on the relationship between healthy lifestyle components and cardiometabolic variables in adolescents. The main findings indicated that the adoption of a greater number of healthy lifestyle habits was inversely associated with SBP among adolescents with general or central obesity. Additionally, the adoption of healthy lifestyle habits was inversely associated with CRP in adolescents with abdominal obesity. Furthermore, adopting healthy lifestyle habits was positively associated with HDL-C values and inversely related to TRG levels.

Although studies aimed at investigating the possible moderating effect of body fat distribution on the relationship between healthy lifestyle habits (physical activity, diet, low alcohol consumption, and non-smoking) and cardiometabolic indicators in adolescents have not been identified, studies conducted with individuals with different health conditions and/or in different age groups have indicated promising results from the adoption of healthy habits as a strategy to reduce cardiometabolic risk [35,36,37]. In a study conducted with 862 adults in Brazil (39.3 ± 11.4 years, 46.4% men) [36], adherence to healthy lifestyle recommendations (physical activity, diet, low alcohol consumption, and not smoking) moderated the relationship between intima–media thickness and glycated hemoglobin among males with cardiovascular disease or its risk factors. In addition, the same study [36] identified that the lower SBP values observed among women without cardiovascular disease or its risk factors were moderated by adherence to these same healthy habits. While these findings in adults provide a useful comparative framework, it is important to note that cardiometabolic responses in adolescents may differ due to ongoing growth and pubertal development; however, similar moderation studies specifically in pediatric populations remain scarce. Another study [37], conducted with children and adolescents with congenital heart disease (n = 227—median age: 10.02 [IQR: 7.08–13.02] years), identified higher chances of elevated body fat mass (OR: 2.52; 95% CI: 1.05–6.04), central obesity (OR: 4.83; 95% CI: 1.90–12.23), and increased intima–media thickness (OR: 2.20; 95% CI: 1.05–4.62) in those who adopted unhealthy lifestyle habits (i.e., high sedentary behavior and low levels of physical activity) compared to those who adopted healthy habits (i.e., low sedentary behavior and healthy eating habits). Furthermore, a study conducted with the participation of adolescents (n = 1513; 12.5–17.5) from 10 cities in Europe [35] identified that the simultaneous adoption of a healthy diet and physical activity was associated with healthier results regarding the body fat index and lipids (i.e., TG, HDL-C, and CHOL/HDL-C ratio).

The potential mechanisms underlying the effect of adopting different healthy lifestyle habits on reducing SBP and CRP values in adolescents with general and central obesity (CRP only) may be related to the combined cardiometabolic health benefits of each habit: (i) Engaging in physical activity is directly linked to reductions in arterial stiffness, decreased peripheral sympathetic nervous activity, and increased nitric oxide release in the vessels, all of which contribute to lower blood pressure levels [38]. Although the physiological mechanisms underlying the reduction in CRP values associated with physical activity remain not fully elucidated, it is hypothesized that this relationship may be related to a reduction in interleukin 6 (IL-6) values [39]. Since IL-6 was not measured in the present study, this remains a speculative mechanism based on the previous literature. Regular physical activity is associated with decreased IL-6, a cytokine produced in response to distinct acute and chronic inflammatory conditions (e.g., bacterial, viral, or fungal infections, rheumatic diseases, malignancies, and tissue injury and necrosis), which in turn triggers the synthesis of CRP and fibrinogen by the liver [39]. (ii) Additionally, adopting a balanced diet, which includes an increased intake of different vitamins and minerals, can contribute to reduced oxidative stress and a lower low-grade inflammatory status. This dietary approach can lead to decreased CRP levels and positively influence cytokines secreted by adipose tissue, such as adiponectin, which is often reduced in people with obesity [40]. A lower concentration of adiponectin is associated with improved vascular endothelial function and enhanced nitric oxide production, which contributes to a reduction in blood pressure levels [41]. (iii) Additionally, a dose–response relationship may exist between alcohol consumption and blood pressure, with higher doses of alcohol potentially exerting prolonged effects [42]. Acute alcohol consumption decreases the levels of hydroxyeicosatetraenoic acid (20-HETE), a signaling molecule that affects the cardiovascular system through various mechanisms, including vasoconstriction and the inhibition of sodium reabsorption in the proximal and distal renal tubules [43]. Furthermore, alcohol consumption is known to contribute to increased concentrations of pro-inflammatory cytokines (i.e., low-grade inflammation), typically preceding the synthesis of other cytokines [44]. (iv) The harmful relationship between smoking and blood pressure, although well established in the adult population, requires more evidence from studies with representative samples of children and adolescents [45]. This is because the results of surveys conducted with the pediatric population and summarized in a systematic review study with meta-analysis indicated no association between active or passive smoking and the presence of hypertension [45]. In contrast to the aforementioned findings, a study involving 8520 youths aged 8 to 19 years, representing 41 million children and adolescents in the USA, identified a 31% increased likelihood of elevated blood pressure among those exposed to any form of tobacco compared to those who were not exposed [46]. The biological plausibility for the association between smoking and elevated blood pressure is supported by both acute and chronic effects. Acutely, smoking stimulates the sympathetic nervous system, leading to increased blood pressure [47]. Chronic smoking contributes to endothelial dysfunction and vasculopathy [48]. Additionally, smoking induces inflammatory responses through the promotion of oxidative stress, increased coagulation and platelet aggregation, and the impairment of flow-mediated arterial function, which may affect the measurement of CRP values [49]. While the overall trend indicated a reduction in cardiometabolic risk markers with a higher number of healthy habits, some outcomes exhibited non-linear patterns in intermediate categories (one and two habits). These fluctuations suggest a threshold effect, where the most substantial benefits, particularly for SBP and inflammatory markers, are more evident upon the adoption of multiple concurrent healthy behaviors in the presence of obesity.

Regardless of obesity or body fat, the adoption of different healthy lifestyle habits was positively associated with HDL-C and inversely associated with TRGs in adolescents. These results are consistent with health guidelines [1,3], which recommend the integration of different healthy habits practices as a general strategy for improving lipid profiles [1,3]. Such habits are known to promote one another [50]. Specifically, regular physical activity and a healthy diet individually contribute to improvement in the serum lipid profile [50], whereas excessive smoking and alcohol consumption are linked to adverse lipid and lipoprotein levels [48,51]. Therefore, it is hypothesized that complex and additive interactions between these lifestyle components may explain the positive association with HDL-C and the negative association with TRGs in adolescents. Furthermore, understanding which specific behaviors are most prevalent among adolescents with obesity is crucial. In our sample, the adoption of lifestyle habits by adolescents with obesity did not significantly differ from that of their normal-weight peers (Figure 2). This suggests that the observed cardiometabolic benefits occur across all adiposity levels, even without immediate weight loss, although these benefits are more pronounced in the presence of excess weight.

This study has several notable strengths, including the investigation of obesity using distinct methods and the reliance on measured rather than self-reported clinical data. However, certain limitations should be acknowledged: (i) We must acknowledge that the high attrition rate from the original sample to the sub-sample with blood data may introduce selection bias, as adolescents who volunteer for blood collection might have different health-seeking behaviors. (ii) Furthermore, the reliance on a single-item “balanced diet” question from the Fantastic Lifestyle questionnaire captures a global perception of dietary quality rather than precise nutrient intake. This perception may be influenced by individuals’ nutrition literacy and subjective interpretation of portion sizes. While this limits our ability to discuss specific macro- or micronutrient effects, previous validation studies for this instrument in Brazil show it to be a reliable proxy for general health-related behavioral patterns in adolescents [18]. (iii) Although heterogeneity analyses in this study did not indicate a moderating effect of sex on the tested associations, it is biologically plausible that men may have a greater distribution of central fat relative to overall fat mass compared to women. This could potentially result in different outcomes when analyzed by sex. (iv) The lack of information regarding the temporality of the presence of obesity and/or body fat in study participants is a limitation since body fat in the long term (when compared to the short term) tends to be more harmful to cardiometabolic health [1]. (v) The use of lifestyle information obtained through self-reports is a limitation of this study. Specifically, regarding smoking habits, the instrument used primarily focused on conventional tobacco use. Given that data collection occurred in 2019, the assessment did not explicitly distinguish between conventional cigarettes and electronic nicotine delivery systems (e-cigarettes/vaping). Although vaping was less prevalent among Brazilian adolescents at the time of collection compared to current trends, the lack of specific data on other nicotine sources should be considered when interpreting the “non-smoking” habit results. (vi) The cross-sectional design of the analyses cannot be used to infer causal associations (i.e., number of healthy lifestyle components that lead to better outcomes, or vice versa, or even mutual influence). (vii) The analytical sample (n = 340) is significantly smaller than the initial estimated sample size. While post hoc power calculations indicated sufficient power for the primary associations tested, we must acknowledge that the substantial attrition rate (from 886 interviewed to 340 with blood data) compromises the representativeness of the findings. Consequently, the results may not be fully generalizable to the entire adolescent population of São José, as the sub-sample may represent a group with different health behaviors or sociodemographic characteristics. In addition to the described limitations, the use of different cut-off points to classify adherence to the recommendations for physical activity, alcohol consumption, and cigarette use for individuals aged up to 17 years and those aged 18 years or older is considered a limitation of the present study. (viii) With regard to adherence to physical activity recommendations, although it may seem unlikely that the dose of physical activity at which health benefits occur for those over 18 years of age drastically differs from the recommended levels of physical activity for individuals aged 18 or 19 years (included in the present study) [52], the use of different metrics may affect the ability to adequately monitor adherence to physical activity across the entire age range of adolescents investigated in this study [52]. Regarding alcohol consumption and cigarette use, since in Brazil only adolescents aged 18 years and older are authorized to purchase and consume them, meaning they can use them legally, it is possible that the prevalence results for these habits may have been influenced by the different contexts in which these adolescents are inserted in terms of legality. Furthermore, while our binary classification focused on high-risk patterns (binge drinking), it is important to acknowledge that lower-dose frequent consumption also warrants attention in comprehensive lifestyle assessments, as it may contribute to long-term cardiometabolic risk. (ix) Regarding maturational status, although self-assessment is a proxy for clinical examination, this method has been previously validated for the Brazilian population and is a standard procedure in large-scale epidemiological surveys. To reduce potential misclassification and ensure that the adjustment for sexual maturation effectively controlled for its robust influence on cardiometabolic outcomes without over-parametrizing our models, we employed collapsed pubertal categories (pre-pubertal, pubertal, and post-pubertal). This categorization minimizes the subjective bias of self-reports while still accounting for the essential biological variance associated with pubertal development.

## 5. Conclusions

The present study identified that the adoption of multiple healthy lifestyle habits was associated with a dose–response trend in cardiometabolic risk markers, albeit with small effect sizes, among adolescents with obesity in different regions of the body. Specifically, multiple healthy lifestyle habits were inversely associated with SBP in adolescents with general and central obesity and inversely associated with CRP levels among those with central obesity. These findings suggest a potential role for adhering to comprehensive healthy lifestyle habits within the context of strategies designed to address the risk of cardiometabolic diseases in adolescents, especially those with general and central obesity. Although our cross-sectional data do not allow for the evaluation of interventions or behavior change, these results provide a hypothesis-generating basis to suggest that intervention programs focusing on the simultaneous adoption of multiple healthy habits, rather than isolated behaviors, may warrant further investigation. In this context, adolescents with obesity who exhibit minimal or no adherence to healthy habits could be considered a priority group for future targeted support and guidance in adopting healthier lifestyles.

## Figures and Tables

**Table 1:** General information on the entire sample (n = 340) and stratified according to sex (male, n = 153; female, n = 187).

Variable	Total n = 340 (100%)	Male n = 153 (45.2%)	Female n = 187 (54.8%)	*p* Value *
**Continuous variables (mean ± SD)**				
Age (years)	16.6 ± 1.0	16.8 ± 1.0	16.5 ± 1.0	0.010
BMI (kg.m^−2^)	22.9 ± 4.4	22.7 ± 4.3	23.1 ± 4.5	0.570
WC (cm)	72.4 ± 9.0	74.7 ± 7.7	70.5 ± 9.5	<0.001
Body fat (%)	21.0 ± 7.4	15.8 ± 5.6	25.3 ± 5.8	<0.001
**Categorical variables, n (%)**				
**Socioeconomic level**				0.372
Classes D–E	5 (1.5)	3 (1.9)	2 (1.1)	
Class C2	30 (9.6)	8 (7.1)	22 (11.8)	
Class C1	81 (23.1)	37 (23.0)	44 (23.5)	
Class B2	162 (47.3)	75 (47.8)	87 (46.9)	
Class B1	43 (12.9)	22 (15.4)	21 (10.8)	
Class A	19 (5.6)	8 (4.8)	11 (5.9)	
**Maturational status**				0.839
Pre-pubertal	36 (10.5)	17 (11.6)	19 (9.6)	
Pubertal	251 (74.4)	114 (74.2)	137 (74.6)	
Post-pubertal	53 (15.1)	22 (14.2)	31 (15.8)	
**Healthy lifestyle habits**				
Healthy dietary habits	41 (12.2)	27 (18.1)	14 (7.3)	0.004
Low alcohol consumption	197 (57.7)	90 (58.0)	107 (54.5)	0.766
Non-smoking	282 (82.5)	130 (83.7)	152 (81.4)	0.369
Physically active	38 (11.9)	24 (15.7)	14 (8.8)	0.017
**Adiposity status**				
Overweight/obesity ^a^	98 (29.9)	35 (22.8)	63 (35.9)	0.029
Abdominal obesity ^b^	74 (22.3)	32 (21.6)	42 (22.8)	0.731
High body fat ^c^	84 (25.1)	32 (20.8)	52 (28.5)	0.143

BMI: body mass index; WC: waist circumference. * T test for continuous data and chi-square test for categorical data; ^a^: considering those classified as overweight or obese based on body mass index (kg/m^2^); ^b^: based on waist circumference (cm); ^c^: based on body fat percentage assessed by triceps and subscapular skinfolds.

**Table 2:** Adjusted ^a^ association between number of healthy lifestyle habits and cardiometabolic variables (n = 340).

		Number of Healthy Lifestyle Recommendations					
Outcomes	Overall, Mean ± SD	None, Mean (SE)	1, β (SE)	2, β (SE)	3+, β (SE)	*p* Value	Partial η^2^
**SBP (mm Hg)**	111.1 ± 14.8	107.5 (1.7)	4.2 (3.5)	4.9 (4.1)	−0.1 (1.1)	0.854 ^e,f^	0.015 ^e^; 0.019 ^f^
**DBP (mm Hg)**	68.2 ± 9.7	67.6 (1.7)	−0.3 (0.9)	0.6 (1.0)	2.2 (2.1)	0.464	
**CHOL (mg/dL)**	146.1 ± 33.2	146.0 (0.9)	3.9 (1.5)	3.5 (4.0)	−5.7 (4.7)	0.489 ^f^	0.022 ^f^
**HDL-C (mg/dL)**	47.2 ± 11.3	47.3 (0.4)	0.3 (0.6)	0.7 (1.0)	1.2 (1.0)	0.038	
**LDL-C (mg/dL)**	89.3 ± 28.2	86.9 (0.6)	4.4 (2.4)	3.8 (4.0)	−6.6 (7.0)	0.495 ^e^	0.014 ^e^
**lnTRG**	57.0 [45.0–81.0] ^b^	62.3 (1.1) ^c^	0.98 (0.16) ^d^	0.95 (0.15) ^d^	0.89 (0.08) ^d^	0.022 ^g^	0.005 ^g^
**FG (mg/dL)**	80.3 ± 7.1	77.6 (2.3)	2.7 (1.8)	3.4 (1.8)	2.3 (1.5)	0.145	
**lnHOMA-IR**	1.77 [1.30–2.50] ^b^	1.65 (1.00) ^c^	1.07 (0.05) ^d^	1.09 (0.04) ^d^	1.03 (0.06) ^d^	0.482	
**lnCRP**	1.20 [0.70–2.10] ^b^	1.12 (1.09) ^c^	1.24 (0.34) ^d^	1.04 (0.19) ^d^	1.34 (0.32) ^d^	0.587 ^f^	0.015 ^f^

SD: standard deviation; SE: standard error; SBP: systolic blood pressure; DBP: diastolic blood pressure; CHOL: cholesterol; HDL-C: high-density lipoprotein cholesterol; LDL-C: low-density lipoprotein cholesterol; lnTRG: natural logarithm of triglycerides; lnHOMA-IR: natural logarithm of homeostatic model assessment index; FG: fasting glucose; lnCRP: natural logarithm of high-sensitivity C-reactive protein; η^2^: partial eta-squared. ^a^: Adjusted for sex, age, socioeconomic level, and sexual maturity. ^b^: Median and interquartile range of the original variable (natural numbers). ^c^: Exponential of the predicted parameters obtained from adjusted regression models based on the natural logarithm of the corresponding outcome. ^d^: Results for log-transformed variables (lnTRG, lnHOMA-IR, and lnCRP) are expressed as exponentials of the regression coefficients (*e*^β^). These should be interpreted as the proportional change in the geometric mean compared to the reference group (None). For example, a value of 1.07 represents a 7% increase, while a value of 0.93 represents a 7% decrease in the outcome. ^e^: *p* value for the interaction between lifestyle variables and general obesity assessed by BMI (normal weight/overweight + obesity) < 0.10. ^f^: *p* value for the interaction between lifestyle variables and abdominal obesity assessed by waist circumference (no/yes) < 0.10. ^g^: *p* value for the interaction between lifestyle variables and body fat assessed by body fat percentage (normal/excess) < 0.10.

## Data Availability

The data presented in this study are available on request from the corresponding author. The data are not publicly available due to the fact that the data utilized in epidemiological research originate from surveys conducted with human participants, and participants’ data are safeguarded regarding access to this information.

## Supplementary Materials

The following supporting information can be downloaded at https://www.mdpi.com/article/10.3390/healthcare14030328/s1: Figure S1: Adjusted predictions of interaction between healthy lifestyle and body mass index (normal weight; overweight/obesity) when associated with systolic blood pressure; Figure S2: Adjusted predictions of interaction between healthy lifestyle and waist circumference (normal; obesity) when associated with systolic blood pressure; Figure S3: Adjusted predictions of interaction between healthy lifestyle and waist circumference (normal; obesity) when associated with cholesterol; Figure S4: Adjusted predictions of interaction between healthy lifestyle and body mass index (normal weight; overweight/obesity) when associated with LDL cholesterol; Figure S5: Adjusted predictions of interaction between healthy lifestyle and waist circumference (normal; obesity) when associated with C-reactive protein; Figure S6: Adjusted predictions of interaction between healthy lifestyle and body fat (normal; excess) when associated with triglycerides; Table S1: Adjusted results of the association between healthy lifestyle and cardiometabolic indicators and interaction values of obesity (assessed by BMI—normal weight/overweight + obesity) with healthy lifestyle when associated with cardiometabolic indicators; Table S2: Adjusted results of the association between healthy lifestyle and cardiometabolic indicators and interaction values of obesity (assessed by waist circumference—no/yes) with healthy lifestyle when associated with cardiometabolic indicators; Table S3: Adjusted results of the association between healthy lifestyle and cardiometabolic indicators and interaction values of obesity (assessed by body fat—normal/excess) with healthy lifestyle when associated with cardiometabolic indicators.
