# Supplementary material for: Does the Association Between Healthy Lifestyle and Cardiometabolic Variables in Adolescents Depend on Obesity and Its Distribution?
Source: Healthcare (Basel). 2026 Jan 28;14(3):328. doi: 10.3390/healthcare14030328 (PMC12896649; doi:10.3390/healthcare14030328)
Supplement: Supplementary file 1 [file healthcare-14-00328-s001.zip › Supplementary Figure captions.pdf]

Supplementary Figure captions

Supplementary Figure 1. Adjusted predictions of interaction between healthy lifestyle and body mass index (normal weight; overweight/obesity) when associated with systolic blood pressure.

Supplementary Figure 2. Adjusted predictions of interaction between healthy lifestyle and waist circumference (normal; obesity) when associated with systolic blood pressure.

Supplementary Figure 3. Adjusted predictions of interaction between healthy lifestyle and waist circumference (normal; obesity) when associated with cholesterol.

Supplementary Figure 4. Adjusted predictions of interaction between healthy lifestyle and body mass index (normal weight; overweight/obesity) when associated with LDL - Cholesterol.

Supplementary Figure 5. Adjusted predictions of interaction between healthy lifestyle and waist circumference (normal; obesity) when associated with C-reactive protein.

Supplementary Figure 6. Adjusted predictions of interaction between healthy lifestyle and body fat (normal; excess) when associated with triglycerides.
