# Supplementary material for: Does the Association Between Healthy Lifestyle and Cardiometabolic Variables in Adolescents Depend on Obesity and Its Distribution?
Source: Healthcare (Basel). 2026 Jan 28;14(3):328. doi: 10.3390/healthcare14030328 (PMC12896649; doi:10.3390/healthcare14030328)
Supplement: Supplementary file 1 [file healthcare-14-00328-s001.zip › Supplementary Tables.pdf]

Supplementary Table 1. Adjusted[a] results of the association between healthy lifestyle and cardiometabolic indicators and interaction values of obesity (assessed by BMI - normal weight/overweight + obesity) with healthy lifestyle when associated with cardiometabolic indicators.

	Main effect		Healthy lifestyle * BMI			
**Outcomes**	**β (SE)**	**p value**	**β**	**95% CI**	**p value**	**η^2**
**SBP (mm Hg)**	0.66 (1.09)	0.543	-2.80	-7.04; -0.82	0.019	0.028
**DBP (mm Hg)**	0.27 (0.77)	0.727	1.03	-1.93; 4.00	0.495	0.009
**Chol (mg/dL)**	-0.04 (2.48)	0.986	-2.27	-11.90; 7.36	0.643	0.012
**HDL-C (mg/dL)**	0.47 (0.83)	0.572	0.97	-2.27; 4.21	0.555	0.006
**LDL-C (mg/dL)**	-1.02 (2.17)	0.683	-3.59	-6.62; -0.56	0.030	0.024
**lnTRG**	0.97[b] (0.03)	0.438	0.96[b]	0.84; 1.09	0.592	0.011
**FG (mg/dL)**	0.69 (0.57)	0.225	-0.15	-2.35; 2.04	0.890	0.005
**lnHOMA-IR**	1.01[b] (0.04)	0.759	1.00[b]	0.85; 1.18	0.953	0.011
**lnCRP**	1.08[b] (0.07)	0.283	0.74[b]	0.54; 1.02	0.069	0.020

β: Beta coefficient; η^2: Partial Eta Squared (effect size); CI: Confidence interval; BMI: Body Mass Index; SBP: Systolic blood pressure; DBP: Diastolic blood pressure; CHOL: Cholesterol; HDL-C: High-density lipoproteins cholesterol; LDL-C: Low-density lipoproteins cholesterol; lnTRG: Natural logarithm of triglycerides; lnHOMA-IR: Natural logarithm of homeostatic model assessment index; FG: Fasting glucose; lnCRP: Natural logarithm of high-sensitivity C-reactive protein.

a: Adjusted for sex, age, socioeconomic level, and sexual maturity;

b: Results expressed in the exponential form and should be interpreted as a risk ratio compared with the mean value observed among those who did not adopt any lifestyle habits.

Supplementary Table 2. Adjusted[a] results of the association between healthy lifestyle and cardiometabolic indicators and interaction values of obesity (assessed by waist circumference – no/yes) with healthy lifestyle when associated with cardiometabolic indicators.

	Main effect		Healthy lifestyle * Waist circumference			
**Outcomes**	**β (SE)**	**p value**	**β**	**95% CI**	**p value**	**η^2**
**SBP (mm Hg)**	0.40 (1.05)	0.700	-2.39	-6.99; -1.31	0.030	0.016
**DBP (mm Hg)**	0.28 (0.74)	0.962	1.22	-2.02; 4.45	0.460	0.016
**Chol (mg/dL)**	-0.44 (2.40)	0.853	-1.25	-2.99; -0.41	0.026	0.027
**HDL-C (mg/dL)**	0.52 (0.82)	0.522	0.97	-2.57; 4.52	0.589	0.008
**LDL-C (mg/dL)**	-0.44 (2.10)	0.833	-3.34	-12.45; 5.77	0.471	0.015
**lnTRG**	0.96 (0.03)[b]	0.201	1.02[b]	0.89; 1.17	0.744	0.029
**FG (mg/dL)**	0.53 (0.55)	0.335	0.54	-1.84; 2.92	0.655	0.006
**lnHOMA-IR**	1.01 (0.03)[b]	0.771	1.00[b]	0.84; 1.20	0.968	0.001
**lnCRP**	1.06 (0.08)[b]	0.374	0.75[b]	0.57; 0.99	0.045	0.027

β: Beta coefficient; η^2: Partial Eta Squared (effect size); CI: Confidence interval; SBP: Systolic blood pressure; DBP: Diastolic blood pressure; CHOL: Cholesterol; HDL-C: High-density lipoproteins cholesterol; LDL-C: Low-density lipoproteins cholesterol; lnTRG: Natural logarithm of triglycerides; lnHOMA-IR: Natural logarithm of homeostatic model assessment index; FG: Fasting glucose; lnCRP: Natural logarithm of high-sensitivity C-reactive protein.
a: Adjusted for sex, age, socioeconomic level, and sexual maturity;
b: Results expressed in the exponential form and should be interpreted as a risk ratio compared with the mean value observed among those who did not adopt any lifestyle habits.

Supplementary Table 3. Adjusted[a] results of the association between healthy lifestyle and cardiometabolic indicators and interaction values of obesity (assessed by body fat – normal/excess) with healthy lifestyle when associated with cardiometabolic indicators.

	Main effect		Healthy lifestyle * Body fat			
**Outcomes**	**β (SE)**	**p value**	**β**	**95% CI**	**p value**	**η^2**
**SBP (mm Hg)**	0.87 (1.10)	0.433	-3.03	-4.25; 1.18	0.157	0.010
**DBP (mm Hg)**	0.18 (0.77)	0.813	1.51	-1.43; 4.45	0.314	0.003
**Chol (mg/dL)**	-1.12 (2.49)	0.650	2.13	-7.38; 11.65	0.659	0.006
**HDL-C (mg/dL)**	0.92 (0.84)	0.276	-1.12	-4.33; 2.09	0.492	0.014
**LDL-C (mg/dL)**	-1.43 (2.17)	0.511	1.94	-6.36; 10.24	0.646	0.008
**lnTRG**	0.95 (0.03)[b]	0.137	1.07[b]	1.00; 1.21	0.047	0.014
**FG (mg/dL)**	0.72 (0.57)	0.205	-0.20	-2.39; 1.98	0.853	0.010
**lnHOMA-IR**	1.00 (0.04)[b]	0.834	1.04[b]	0.89; 1.23	0.564	0.018
**lnCRP**	1.04 (0.07)[b]	0.600	0.93[b]	0.70; 1.24	0.629	0.007

β: Beta coefficient; η^2: Partial Eta Squared (effect size); CI: Confidence interval; SBP: Systolic blood pressure; DBP: Diastolic blood pressure; CHOL: Cholesterol; HDL-C: High-density lipoproteins cholesterol; LDL-C: Low-density lipoproteins cholesterol; lnTRG: Natural logarithm of triglycerides; lnHOMA-IR: Natural logarithm of homeostatic model assessment index; FG: Fasting glucose; lnCRP: Natural logarithm of high-sensitivity C-reactive protein.

a: Adjusted for sex, age, socioeconomic level, and sexual maturity;

b: Results expressed in the exponential form and should be interpreted as a risk ratio compared with the mean value observed among those who did not adopt any lifestyle habits.
