# Supplementary figures and images for: Does the Association Between Healthy Lifestyle and Cardiometabolic Variables in Adolescents Depend on Obesity and Its Distribution?
Source: Healthcare (Basel). 2026 Jan 28;14(3):328. doi: 10.3390/healthcare14030328 (PMC12896649; doi:10.3390/healthcare14030328)

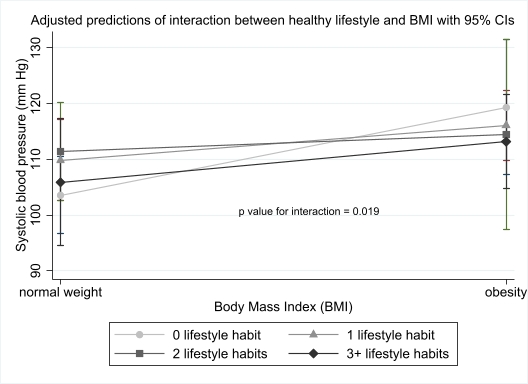

Supplement: Supplementary file 1 [file healthcare-14-00328-s001.zip › Supplementary Figure 1.jpg]

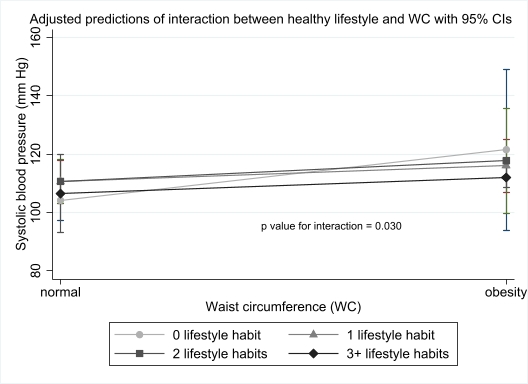

Supplement: Supplementary file 1 [file healthcare-14-00328-s001.zip › Supplementary Figure 2.jpg]

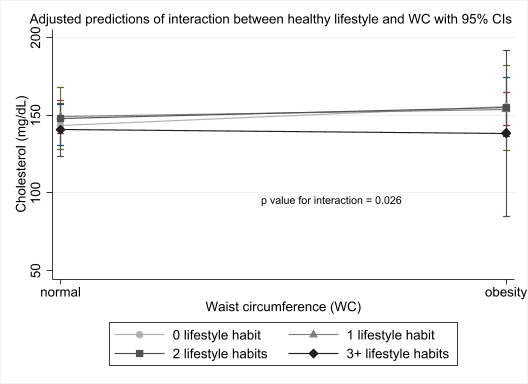

Supplement: Supplementary file 1 [file healthcare-14-00328-s001.zip › Supplementary Figure 3.jpg]

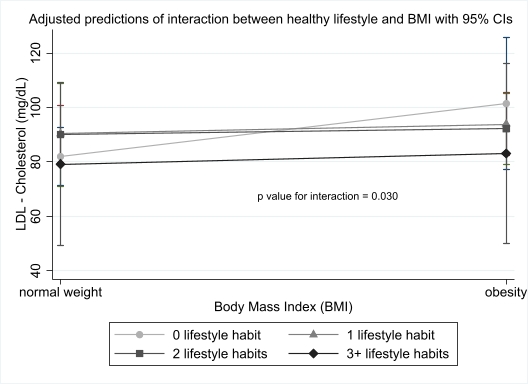

Supplement: Supplementary file 1 [file healthcare-14-00328-s001.zip › Supplementary Figure 4.jpg]

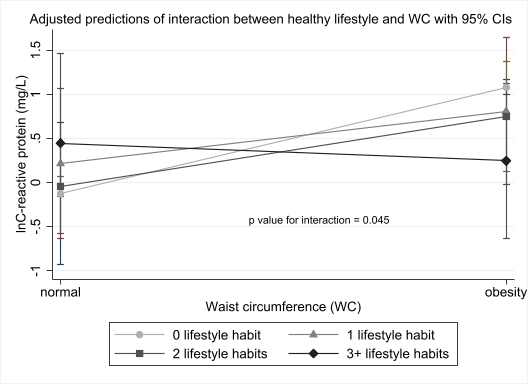

Supplement: Supplementary file 1 [file healthcare-14-00328-s001.zip › Supplementary Figure 5.jpg]

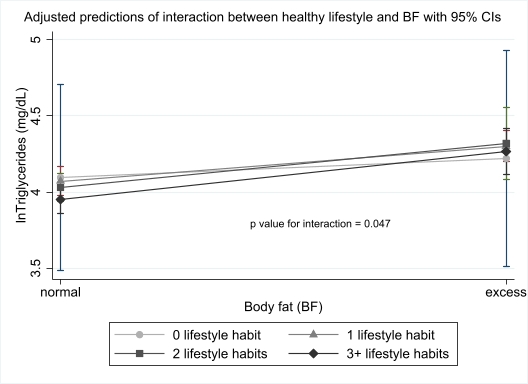

Supplement: Supplementary file 1 [file healthcare-14-00328-s001.zip › Supplementary Figure 6.jpg]
